# Impact of different dietary fat sources on blood pressure in Chinese adults

**DOI:** 10.1371/journal.pone.0247116

**Published:** 2021-03-08

**Authors:** Qiaoling Liu

**Affiliations:** Faculty of Epidemiology and Population Health, London School of Hygiene & Tropical Medicine, London, United Kingdom; The Ohio State University, UNITED STATES

## Abstract

**Objectives:**

To investigate the association between the source of dietary fat and blood pressure for Chinese people aged between 30–59 with the aim of elucidating methods of hypertension prevention.

**Design:**

Prospective cohort study using national survey data.

**Participants:**

1,104 adults aged between 30–59 with normal blood pressures in 2006 were included in the study. Adults with history of prehypertension, hypertension, or were taking hypertension drugs in 2006 were excluded. Participants with implausibly high or low daily total energy intakes (<600 kcal/d or >5000 kcal/d) were excluded. Pregnant women, breastfeeding women, and people with motor impairment were excluded.

**Results:**

People with abnormal blood pressure ingested a lower percent of dietary fat taken from seafood (*P* < 0.001) and a higher percent of dietary fat taken from fast food (*P* < 0.001). Dietary fat obtained from seafood and dairy products can be protective against abnormal blood pressure with a RR = 0.01 (95% CI: 0.001 to 0.25; *P* = 0.004) for seafood, and RR = 0.14 (95% CI: 0.04 to 0.44; *P* = 0.001) for egg, milk, and dairy products.

**Conclusion:**

Seafood, egg, milk, and dairy products can be recommended as sources of dietary fat to reduce the incidence of hypertension and prehypertension while fast food should be avoided.

## Introduction

China has been suffering from a continuous increase in the incidence of hypertension in recent decades. The 6 national hypertension surveys conducted between 1958 and 2015 reflected a continuous increase in the prevalence of hypertension. The latest China Hypertension Survey conducted between 2012 and 2015 covers 451,755 adults from 31 provinces [1]. The survey found that 27.9% of Chinese adults have hypertension. The National Center for Cardiovascular Diseases estimates the total number of hypertension patients is 245 million in 2018 [2]. A growth of hypertension incidence among middle-aged people has been observed [3]. The deteriorated life quality and economic burden have made the prevention of hypertension a meaningful public health issue in China.

It is clear that the prevalence of hypertension is different among ages, genders, and geographic regions [4]. Risk factors for hypertension include insufficient physical activity, obesity, and poor diet [5]. The necessity of food for survival makes the diet one of the factors that affects all people. Preventing hypertension through dietary adjustment is a method that fits the philosophy of public health which aims to promote the health of a population.

Dietary fat is a macronutrient that serves as a main energy source for human activities while also transporting the fat-soluble vitamins and constructing cell membranes. Nevertheless, excessive ingestion of dietary fat may lead to overweight and higher risk of disease occurrence. Dietary fat includes saturated fat, trans fat, monounsaturated fat, and polyunsaturated fat. Saturated and trans fats can be found in fatty meats and fried foods. They are generally believed to be harmful to the human body. Unsaturated fats, both monounsaturated and polyunsaturated fats, exist widely in fish and other seafoods. The majority of hypertension patients have been diagnosed with high cholesterol [6] which is positively correlated with systolic blood pressure (SBP) [7]. Replacing saturated fat and carbohydrate with unsaturated fat can lower the risk of cardiovascular disease [8]. Studies have focused on the effect of fat type on blood pressure (BP) but not on the source of dietary fat. Using data from the China Health and Nutrition Survey (CHNS), we conducted a cohort study on the source of dietary fat and BP for Chinese people aged between 30–59 with the aim of elucidating methods of hypertension prevention.

## Materials and methods

### Study population

The CHNS is co-hosted by the Carolina Population Center at the University of North Carolina at Chapel Hill and the National Institute for Nutrition and Health at the Chinese Center for Disease Control and Prevention. The objective is to examine the effects of health and nutrition policies and programs implemented by different levels of governments. The CHNS study was approved by the institutional review boards of the University of North Carolina at Chapel Hill and the National Institute for Nutrition and Health, Chinese Center for Disease Control and Prevention. (Reference No. 201524).

This study used survey data from CHNSs conducted in 2006, 2009, and 2011. All the nine provinces available in the three surveys were included, namely, Liaoning, Heilongjiang, Jiangsu, Shandong, Henan, Hubei, Hunan, Guangxi, and Guizhou. The number of provinces in CHNS study is fewer than the total number of provinces in China. However, it should be noted that CHNS was not designed to represent China, but with an objective to reflect a broad range of socioeconomic and demographic contexts using random sampling methods. For the detailed sampling method and survey design of the CHNS, please refer to other reports [9].

Adults aged between 30–59 with normal BPs in 2006 were included in the study. None of the subjects had any history of prehypertension, hypertension, or were taking hypertension drugs in 2006. Participants with implausibly high or low daily total energy intakes (<600 kcal/d or >5000 kcal/d) were excluded. Pregnant women, breastfeeding women, and people with motor impairment were excluded. A total of 1,104 individuals entered this cohort study. The follow-up time was five years.

### Assessment and categorization of blood pressure

This research used BP data from the CHNS. Standard mercury sphygmomanometer (sub division:2mmHg, measuring scope: 0-300mmHg) and stethoscope were used to measure blood pressure. Each sphygmomanometer was calibrated before measuring. A detailed description of blood pressure measuring procedure can be found in the survey’s training material [10]. All the surveys followed the same procedure.

SBP and diastolic blood pressure (DBP) were measured three times with a 2-minute interval between each test. The average of the second and third test results was used to identify the individual’s BP status.

Individuals were categorized into three different BP status groups according to the 2018 Chinese Guidelines for Prevention and Treatment of Hypertension [3]. Normal BP was defined as SBP < 120 mmHg and DBP < 80 mmHg, prehypertension was either SBP between 120–139 mmHg or DBP between 80–89 mmHg, and hypertension was defined as either SBP ≥ 140 mmHg or DBP ≥ 90 mmHg.

### Assessment of dietary fat intake

In each CHNS survey, an individual’s dietary intake was measured for three consecutive days by asking the individual to report all the food consumed on a 24-hour recall basis. In our research, every individual has completed a three consecutive days dietary measurement in 2006, 2009, and 2011 respectively. A total of nine days’ dietary intake was measured.

Each reported food was assigned to a food code and a food type using China Food Composition Table (FCT) 2002 and 2004 [11, 12]. The food code and food type are unique for each food. A food type can be assigned to different foods. In FCT, each food’s fat per 100 edible grams is listed. Therefore, by matching CHNS food code and type with FCT data, we can get the fat per 100 edible grams of each food reported in CHNS. The fat intake from each reported food was then aggregated at the level of food type to calculate an individual’s fat intake from a food type. Then, an individual’s fat intake from a food type was divided by the individual’s total fat intake during the dietary assessment to get the percentage of dietary fat intake of a food type in relation to the total fat intake. Please refer to the S1 File for detailed calculations.

Based on FCT and the dietary fat intake pattern among Chinese adults [13], we selected the following five food types from FCT as our research interests: (1) Meat and meat products, (2) Poultry and poultry products, (3) Egg, milk, and dairy products, (4) Seafood, and (5) Fast food. The coverage and example of FCT food types are listed as Table 1.

**Table 1:** Food type, subtype, and examples from China Food Composition Table 2002 and 2004.

Food type	Food subtype	Example
1. Meat and meat products	Pig, cattle, mutton, ass, horse, others	Beef, pork, lamb, ribeye, lung, heart, liver
2. Poultry and poultry products	Chicken, duck, goose, turkey, others	Drumstick, chicken breast, chicken wing
3. Eggs, milk, and dairy products	Liquid cow’s milk, cow’s milk powder, yogurt, cheese, butter and cream, hen egg, duck egg, goose egg, quail egg	Milk, whole milk powder, yogurt
4. Seafood	Fish, prawn and shrimp, crab, shellfish, others	Grass carp, chub, crucian carp, striped bass, sea crab
5. Fast food	Handy food, convenience food, snack	Hamburger, sandwich, fried chip, fried chicken, instant noodle

### Assessment of other factors

This study assessed a variety of factors including gender, age, living area, geographical area, smoking, drinking, weight, height, waist circumference, marital status, intake of vegetable and fruit, intake of carbohydrates, and the dietary intake ratio of potassium to sodium.

An individual’s living area was categorized as either urban or rural area. Geographical area was divided into Northern China and Southern China by the Qinling-Huaihe Line. Liaoning, Heilongjiang, Shandong, and Henan provinces were labeled as Northern China while Jiangsu, Hubei, Hunan, Guangxi, and Guizhou provinces were assigned as Southern China.

We defined BMI categories using China overweight/obesity medical nutrition treatment expert consensus (2016 edition) [14]. Each individual’s body mass index (BMI) was calculated on each survey date. BMIs from 24–27.9 kg/m^2^ were categorized as overweight while BMIs ≥ 28 kg/m^2^ were defined as obese.

It has been shown that waist circumference is positively correlated with hypertension [15]. For males, a waist circumference ≥ 85 cm was categorized as central obesity. The standard for female central obesity was waist circumference ≥ 80 cm.

Fruit and vegetable consumption is inversely associated with blood pressure [16, 17]. In Dietary Guidelines for Chinese Residents [18], the recommended daily lower limit of fruit is 200 grams, and 300 grams for vegetable. We categorized people to low fruit intake or low vegetable intake if their consumption is below the daily lower limit.

High-carbohydrate diet is associated with high blood pressure [19]. The major sources of refined carbohydrates include refined grain, white flour, and noodle which are also the common staples in Chinese people. We calculated the composition of cereal food in the 1,104 people’s dietary. The top three cereal foods by weight are white rice (66.1%), fine dried noodle (12.8%), and steamed white bun (12.4%). All of them are sources of refined carbohydrates and contributed to an average of 56.0% of an individual’s average daily energy. We conclude the major cereal food in an individual’s daily diet is of refined carbohydrates. In Dietary Guidelines for Chinese Residents [18], the recommended daily upper limit of cereal food is 400 grams. We categorized people to high carbohydrate group if their cereal food consumption exceeds the daily upper limit.

It has been reported that added sugar such as high fructose corn syrup is associated with blood pressure in adults [20]. However, added sugar was not measured in CHNS dietary assessment. We explored the feasibility of evaluating sugar intake by looking into the consumption of sweets, beverage, confectionery, and sweetened preserves. We found only 4.0% of individual dietary assessment in our research reported consumption of sweets, beverage, confectionery, and sweetened preserves. Only 1.1% of individuals consumed these food in more than one CHNS survey. We concluded individual consumption of sweets, beverage, confectionery, and sweetened preserves in our sample is very sporadic and not suitable for analysis. This is our limitation. Detailed information on added sugar consumption will be sought in our future researches.

An individual’s intake of potassium and sodium was measured in CHNS dietary assessment. We used potassium-to-sodium (K-Na) ratio instead of sodium or potassium alone for model adjustments. It has been found while potassium supplementation has a significant effect in reducing BP in hypertensive patients, the increased daily urinary K-Na ratio significantly correlates to BP reduction [21]. The K-Na ratio is a better indicator than potassium or sodium alone in evaluating blood pressure outcomes and hypertension incidence [22]. We calculated K-Na ratio of an individual’s dietary by dividing the total intake of dietary potassium by the total intake of dietary sodium.

### Statistical analyses

Based on participants’ BP status in 2011, participants were categorized into two groups, the normal BP group or the abnormal BP group which includes both prehypertension and hypertension. A χ^2^ test was used to compare the characteristics of the two groups. An age- and sex-adjusted panel logistic regression and a fully-adjusted multivariate panel logistic regression were performed to explore the association between dietary fat intake from different food types and BP. Finally, we explored the association between dietary fat intake from different food types on prehypertension group and hypertension group respectively using multivariate logistic regressions.

## Results

A total of 1,104 people were included in this cohort study of which 64.95% was female. Smoking, drinking, BMI, waist circumference, central obesity percentage, SBP, and DBP all increased over time in Table 2. By 2011, the average of all participants’ SBP was 116.65 mmHg and DBP was 75.90 mmHg, an roughly 11.02% increase from the initial average of SBP and DBP in 2006.

**Table 2:** General characteristics of selected Chinese adults (n = 1,104) aged between 30–59 in each survey round of the China Health and Nutrition Survey, 2006–2011.

	2006		2009		2011	
	n or Mean	% or SE	n or Mean	% or SE	n or Mean	% or SE
**Gender**						
**Male**	387	35.05	387	35.05	387	35.05
**Female**	717	64.95	717	64.95	717	64.95
**Age**						
**30~39**	328	29.71	215	19.47	145	13.13
**40~49**	415	37.59	418	37.86	431	39.04
**50~59**	361	32.70	397	35.96	387	35.05
**60~69**	0	0.00	74	6.70	141	12.77
**Marital Status**						
**Married**	1048	94.93	1044	94.57	1032	93.48
**Single**†	56	5.07	60	5.43	72	6.52
**Smoke**						
**Yes**	278	25.18	313	28.35	335	30.34
**No**	826	74.82	791	71.65	769	69.66
**Drink**						
**Yes**	298	26.99	416	37.68	469	42.48
**No**	806	73.01	688	62.32	635	57.52
**Dietary K-Na Ratio**‡	4.90	3.74	4.32	3.31	4.15	3.36
**BMI**‡	22.40	2.75	22.80	3.02	23.20	3.53
**BMI Level**						
**Normal**	824	74.64	741	67.12	696	63.04
**Overweight**	247	22.37	313	28.35	336	30.43
**Obese**	33	2.99	50	4.53	72	6.52
**Waist Circumference (cm)** ‡	77.93	8.44	80.32	9.03	81.04	9.40
**Central Obesity**						
**Yes**	363	32.88	495	44.84	527	47.74
**No**	741	67.12	609	55.16	577	52.26
**SBP (mmHg)** ‡	105.98	8.16	115.78	13.11	116.65	14.12
**DBP (mmHg)** ‡	69.44	5.82	76.30	8.72	75.90	9.15
**Blood Pressure**						
**Normal**	1104	100.00	781	70.74	629	56.97
**Prehypertension**	0	0.00	220	19.92	302	27.36
**Hypertension**	0	0.00	103	9.33	173	15.67
**Living Area**						
**Urban**	268	24.28	268	24.28	268	24.28
**Rural**	836	75.72	836	75.72	836	75.72
**Geographical Location**						
**Northern China**	326	29.53	326	29.53	326	29.53
**Southern China**	778	70.47	778	70.47	778	70.47

^†^Includes divorced, separated, and widowed cases.

^‡^These data are presented as means with their standard errors; otherwise data are numbers and percentages.

Table 3 shows the general characteristics of participants with normal BP (n = 629) and participants with abnormal BP (n = 475, including prehypertension (n = 302) and hypertension (n = 173) cases) by the end of the cohort duration. There were significant differences in almost all of the characteristics.

**Table 3:** General characteristics of 1,104 participants grouped by blood pressure status by the end of the cohort duration.

	Normal BP (n = 629)		Abnormal BP (n = 475)		P value
	n or Mean	% or SE	n or Mean	% or SE	
**Gender**					<0.001
**Male**	190	30.21	197	41.47	
**Female**	439	69.79	278	58.53	
**Age**					<0.001
**30~39**	97	15.42	48	10.11	
**40~49**	266	42.29	165	34.74	
**50~59**	188	29.89	199	41.89	
**60~69**	78	12.40	63	13.26	
**Marital Status**					0.03
**Married**	590	93.80	442	93.05	
**Single**†	39	6.20	33	6.95	
**Smoke**					<0.001
**Yes**	169	26.87	166	34.95	
**No**	460	73.13	309	65.05	
**Drink**					<0.001
**Yes**	259	41.18	210	44.21	
**No**	370	58.82	265	55.79	
**Dietary K-Na Ratio**‡	4.21	2.95	4.08	3.84	<0.001
**BMI**‡	22.55	3.12	23.95	3.86	<0.001
**BMI Level**					<0.001
**Normal**	438	69.63	258	54.32	
**Overweight**	169	26.87	167	35.16	
**Obese**	22	3.50	50	10.53	
**Waist Circumference (cm)** ‡	79.06	8.85	83.65	9.49	<0.001
**Central Obesity**					<0.001
**Yes**	253	40.22	274	57.68	
**No**	376	59.78	201	42.32	
**SBP (mmHg)** ‡	110.19	10.08	125.21	14.16	<0.001
**DBP (mmHg)** ‡	71.68	6.61	81.42	9.09	<0.001
**Living Area**					0.14
**Urban**	161	25.6	107	22.53	
**Rural**	468	74.4	368	77.47	
**Geographical Location**					<0.001
**Northern China**	154	24.48	172	36.21	
**Southern China**	475	75.52	303	63.79	

Chi-square analysis was conducted to test the existence of significant differences between normal and abnormal BP groups.

^†^Includes divorced, separated, and widowed cases.

^‡^These data are presented as means with their standard errors; otherwise data are numbers and percentages.

Characteristics of dietary fat intake, fruit intake, vegetable intake, refined carbohydrate intake, K-Na ratio, in the normotensive participants and those with abnormal BP are shown in Table 4. No significant differences in the percentage of dietary fat taken from meat, poultry, egg, milk, dairy products, fruit intake, and cereal intake were found between the normal and abnormal BP groups. There is a very weak evidence of association between vegetable intake and blood pressure (P = 0.07). The percentage of fat taken from fast food is 0.0% at the 75th percentile in both groups, which hints that most of participants have not consumed fast food within the dietary assessment period. However, at the 79th percentile, the percentage of fat taken from fast food is 1.9% in abnormal BP group while the percentage remains at 0.0% in normal BP group. Chi-square test indicates there is very strong evidence that the percentage of fat intake from fast food is different between the two groups. We conclude that people with abnormal BP were less likely to obtain fat from seafood, but more likely to obtain fat from fast food. A lower dietary K-Na ratio in abnormal BP group indicates that people with abnormal BP have a higher dietary sodium intake than healthy people.

**Table 4:** Characteristics of dietary fat intake, fruit intake, vegetable intake, refined carbohydrate intake, K-Na ratio among 1,104 participants, grouped by blood pressure status by the end of the cohort duration.

	Normal BP (n = 629)	Abnormal BP (n = 475)	P value
	Median (25%,75%)	Median (25%,75%)	
**Fat intake % of total daily fat intake (Meat and meat products)**	53.11 (20.70, 72.30)	50.08 (20.18, 70.40)	0.20
**Fat intake % of total daily fat intake (Poultry and poultry products)**	0.00 (0.00, 1.41)	0.00 (0.00, 1.67)	0.75
**Fat intake % of total daily fat intake (Egg, milk, and dairy products)**	4.10 (0.00,12.12)	4.61 (0.00, 11.27)	0.57
**Fat intake % of total daily fat intake (Seafood)**	0.00 (0.00, 2.94)	0.00 (0.00, 1.70)	<0.001
**Fat intake % of total daily fat intake (Fast food)**	0.00 (0.00, 0.00)	0.00 (0.00, 0.00)	<0.001
**Weight % in daily dietary (Fruit intake)**	0.00 (0.00, 6.44)	0.00 (0.00, 7.67)	0.15
**Weight % in daily dietary (Vegetable intake)**	0.31 (0.23, 0.39)	0.29 (0.23, 0.38)	0.07
**Weight % in daily dietary (Cereal intake)**	0.40 (0.31, 0.50)	0.40 (0.32, 0.49)	0.72
**Dietary K-Na ratio**	3.76 (2.34, 5.77)	3.42 (1.99, 5.22)	<0.001

Chi-square analysis was conducted to test the existence of significant differences between normal and abnormal BP groups in variables.

Table 5 presents the multivariate-adjusted risk ratio of abnormal BP according to the source of dietary fat. In the age-adjusted and sex-adjusted models, consumption of meat, poultry, and relevant products were not associated with prehypertension and hypertension. Egg, milk, seafood, and relevant products were negatively associated with prehypertension and hypertension. This association remains unchanged in the multivariate-adjusted model.

**Table 5:** Risk ratios and 95% confidence intervals of abnormal BPs by source of dietary fat.

	Risk Ratio	95% CI	P value
**Meat and meat products**			
**Age- and sex-adjusted**†	0.70	0.44, 1.12	0.13
**Multivariate-adjusted**‡	0.73	0.44, 1.21	0.23
**Poultry and poultry products**			
**Age- and sex-adjusted**†	1.70	0.47, 6.15	0.42
**Multivariate-adjusted**‡	1.90	0.47, 7.74	0.37
**Egg, milk, and dairy products**			
**Age- and sex-adjusted**†	0.16	0.05, 0.45	0.001
**Multivariate-adjusted**‡	0.14	0.04, 0.44	0.001
**Seafood**			
**Age- and sex-adjusted**†	0.02	0.002, 0.27	0.003
**Multivariate-adjusted**‡	0.01	0.001, 0.25	0.004
**Fast food**			
**Age- and sex-adjusted**†	1.87	0.74, 4.69	0.18
**Multivariate-adjusted**‡	1.69	0.62, 4.57	0.30

Panel logistic regression was performed in both age-and sex-adjusted model and multivariate-adjusted model.

^†^Age-and sex-adjusted: adjusted for age, interaction of sex and living area (urban male, urban female, rural male, rural female), geographical location (Southern China, Northern China), and dietary K-Na ratio.

^‡^Multivariate-adjusted: adjusted for age, interaction of sex and living area (urban male, urban female, rural male, rural female), geographical location (Southern China, Northern China), marital status, smoking status, drinking status, central obesity (yes or no), BMI level (normal, overweight, obese), fruit intake, vegetable intake, cereal intake, and dietary K-Na ratio.

We used multinomial logistic regression to further examine the associations between dietary fat and subcategories of abnormal BP, namely prehypertension and hypertension in Table 6.

**Table 6:** Risk ratios and 95% confidence intervals for prehypertension and hypertension by the source of dietary fat.

	Prehypertension (n = 302)			Hypertension (n = 173)		
	Risk Ratio	95% CI	P value	Risk Ratio	95% CI	P value
**Meat and meat products**						
**Age- and sex-adjusted**†	0.74	0.45, 1.21	0.23	0.83	0.45, 1.51	0.54
**Multivariate-adjusted**‡	0.73	0.44, 1.20	0.21	0.83	0.44, 1.58	0.57
**Poultry and poultry products**						
**Age- and sex-adjusted**†	3.06	0.75, 12.43	0.12	2.09	0.36, 12.21	0.41
**Multivariate-adjusted**‡	2.48	0.61, 10.01	0.20	1.79	0.32, 10.08	0.66
**Egg, milk, and dairy products**						
**Age- and sex-adjusted**†	0.23	0.07, 0.75	0.02	0.26	0.06, 1.03	0.05
**Multivariate-adjusted**‡	0.21	0.06, 0.71	0.01	0.22	0.05, 0.92	0.04
**Seafood**						
**Age- and sex-adjusted**†	0.05	0.003, 0.80	0.03	0.05	0.002, 1.12	0.06
**Multivariate-adjusted**‡	0.03	0.002, 0.54	0.02	0.02	0.001, 0.73	0.03
**Fast food**						
**Age- and sex-adjusted**†	3.53	1.12, 11.11	0.03	0.76	0.16, 3.65	0.74
**Multivariate-adjusted**‡	3.15	0.98, 10.17	0.06	0.61	0.12, 2.96	0.54

^†^Age-and sex-adjusted: adjusted for age, interaction of sex and living area (urban male, urban female, rural male, rural female), geographical location (Southern China, Northern China), and dietary K-Na ratio.

^‡^Multivariate-adjusted: adjusted for age, interaction of sex and living area (urban male, urban female, rural male, rural female), geographical location (Southern China, Northern China), marital status, smoking status, drinking status, central obesity (yes or no), and BMI level (normal, overweight, obese), fruit intake, vegetable intake, cereal intake, and dietary K-Na ratio.

In all the multivariate-adjusted models, dietary fat obtained from egg, milk, seafood, and their related products are negatively associated to prehypertension and hypertension with strong evidence. Fat intake from fast food has an almost significant evidence showing its association with prehypertension (RR = 3.15, 95% CI 0.98 to 10.17; P = 0.05). The age- and sex-adjusted model shows a strong evidence of positive association between the fat intake from fast food and prehypertension (RR = 3.53, 95% CI 1.12 to 11.11; P = 0.03).

## Discussion

Since the 2000s, the rapid urbanization of China has granted an increase in the living standard to its citizens. Compared to the planned economy of the 1980s, the sufficient food supply in this century has changed people’s dietary structure [23]. The mainstream food pattern featured by vegetables and grains in past decades has gradually shifted towards a high-fat, high-protein, and high-sodium diet. The development of domestic food industry and the entry of Western franchise restaurants into the Chinese market have further motivated people to eat outside.

This research presented a cohort study of 1,104 adults aged between 30–59 and explored the associations between dietary fat intake from different sources and abnormal BP. Our first regression Table 5 identified the obvious inverse association between the dietary fat obtained from egg, milk, dairy products, seafood, and abnormal BP.

The effect of fat in milk and dairy products is controversial. A prospective cohort of 40,526 French women, with an average follow up of 12.2 years reveals the overall consumption of dairy products was not associated with the risk of hypertension. Another study using 2,245 participants of Rotterdam Study shows dairy product intake is negatively related to hypertension for elders [24]. A cross-sectional study on 39,252 adult males in Shanghai, China revealed that people who follow the dietary pattern of “fruit and milk” have lower SBP and DBP [25]. Another study from the Oxford cohort of the European Prospective Investigation into Cancer and Nutrition (EPIC-Oxford) which includes dietary data from 11,004 participants revealed people who favor fish and seafood have lower incidences of hypertension compared to meat-lovers [26]. Our second regression Table 6 evaluated the association between dietary fat intake from different sources and abnormal blood pressure subgroups. Fat from egg, milk, dairy products, and seafood has significant negative association with prehypertension as they have demonstrated in Table 5. It is tempting to conclude that dietary fat intake from eggs, milk, dairy products, and seafood is protective against prehypertension and hypertension.

In Table 6, fast food presents an almost significant positive association with prehypertension (RR = 3.15, 95% CI 0.98 to 10.17; *P* = 0.05), yet the same association has not been observed in hypertensive people. We cannot rule out the possibility of residual confounding and the small number of fast-food lovers. Yet, a proposed explanation to this phenomenon is that prehypertension is the precursor to hypertension, thus risk factors for abnormal BP among normotensive people are more directly associated to prehypertension than hypertension. The annual growth rate of the fast-food industry in China during the survey period is 13% [27]. People who live in regions with more fast food restaurants have higher mortality and admissions for acute coronary syndromes [28]. The high-calorie nature of fast food is more likely to lead to overweight and obesity [29, 30], which increase the risk of abnormal BP. More studies should be conducted to examine the role of fat intake from fast food among prehypertensive and hypertensive people in China.

With the objective of improving fat metabolism and preventing hypertension, we propose the key recommendation for dietary fat refinement should be to increase the share of unsaturated fat intake and not to focus solely on lowering the amount of total dietary fat intake. Our results suggest that a diet in which the fat intake is mainly from seafood, egg, milk, and related products can contribute to the prevention of hypertension for Chinese people. The intake of fatty fast food should be prevented if possible. Also, the intake of sodium should be controlled to lower the risk of hypertension [31, 32] especially under the circumstance that Chinese people are more sensitive to sodium [33].

This study has several limitations. First, we could not completely exclude the possibility of residual confounding due to unmeasured variables such as sleep pattern, mental stress, and family history of hypertension. Second, the 24-hour recall method may underestimate energy and micronutrients intake yet the magnitude of misreporting of energy intake in all the three retrospective dietary assessment methods (24-hour recall, by estimate, and by weight) are similar [34]. Moreover, the individual dietary intake collected in each CHNS survey has been compared to the individual’s household dietary intake data for quality assurance. If significant discrepancies were found, both the individual and the household will be interviewed for re-confirmation. This recall method does not rely on the literacy and numeracy skills of the individual [35]. Therefore, we regard the 24-hour recall method in this survey as a reliable way in evaluating fat intake. Third, we self-designed formulas to calculate the percentage of an individual’s fat intake from a certain food type in relation of the individual’s overall fat intake. While our formulas are simple calculations which are less prone to errors, we have not taking the possible loss of fat in the cooking process into consideration. It means our calculated fat intake might be slightly higher than the exact amount an individual obtains from dietary. Fourth, this study covers a small number of provinces and has not evaluated the usage of flavorings and cooking methods among provinces. The small coverage of CHNS provinces may make our findings less representative. It is known that people in Northern and Southern China have different tastes and cooking preferences. Yet, we generally adjusted geographical interferences and other socioeconomic variables. It would be valuable to study cooking patterns across the provinces in China to explore risk factors of hypertension at a finer granularity.

Nevertheless, this study has several strengths. We analyzed the effect of dietary fat based on food types instead of fat types. People obtain fat by eating food and food is a combination of different types of fat under most circumstances. Therefore, our analysis on fat intake from food type is useful since it evaluates the effect of fat at the level of food. This feature makes our research very useful for health promotion activities. All the participants were normotensive when entering the cohort and had no obvious dietary changes within the survey period, thus possible reverse causation was minimized.

In conclusion, our study presented the relationship between dietary fat from different food types and their associations with abnormal BP. The share of fat ingested from seafood, egg, milk, and dairy products should be increased as an important component of a dietary approach to prevent prehypertension and hypertension.

## Supporting information

S1 File (DOCX)

## References

[1] Wang Z, Chen Z, Zhang L, Wang X, Hao G, Zhang Z, et al. Status of Hypertension in China: Results From the China Hypertension Survey, 2012–2015. Circulation. 2018;137(22):2344–56. Epub 2018/02/17. 10.1161/CIRCULATIONAHA.117.032380 . 29449338

[2] Ma LY, Chen WW, Gao RL, Liu LS, Zhu ML, Wang YJ, et al. China cardiovascular diseases report 2018: an updated summary. J Geriatr Cardiol. 2020;17(1):1–8. Epub 2020/03/07. 10.11909/j.issn.1671-5411.2020.01.001 . 32133031 PMC7008101

[3] Joint Committee for Guideline R. 2018 Chinese Guidelines for Prevention and Treatment of Hypertension-A report of the Revision Committee of Chinese Guidelines for Prevention and Treatment of Hypertension. J Geriatr Cardiol. 2019;16(3):182–241. Epub 2019/05/14. 10.11909/j.issn.1671-5411.2019.03.014 31080465 PMC6500570

[4] Lu J, Lu Y, Wang X, Li X, Linderman GC, Wu C, et al. Prevalence, awareness, treatment, and control of hypertension in China: data from 1.7 million adults in a population-based screening study (China PEACE Million Persons Project). Lancet. 2017;390(10112):2549–58. Epub 2017/11/06. 10.1016/S0140-6736(17)32478-9 . 29102084

[5] Singh S, Shankar R, Singh GP. Prevalence and Associated Risk Factors of Hypertension: A Cross-Sectional Study in Urban Varanasi. Int J Hypertens. 2017;2017:5491838. Epub 2018/01/20. 10.1155/2017/5491838 . 29348933 PMC5733954

[6] Lloyd-Jones D, Adams R, Carnethon M, De Simone G, Ferguson TB, Flegal K, et al. Heart disease and stroke statistics—2009 update: a report from the American Heart Association Statistics Committee and Stroke Statistics Subcommittee. Circulation. 2009;119(3):480–6. Epub 2009/01/28. 10.1161/CIRCULATIONAHA.108.191259 . 19171871

[7] Stamler J, Liu K, Ruth KJ, Pryer J, Greenland P. Eight-year blood pressure change in middle-aged men: relationship to multiple nutrients. Hypertension. 2002;39(5):1000–6. Epub 2002/05/23. 10.1161/01.hyp.0000016178.80811.d9 . 12019283

[8] Liu AG, Ford NA, Hu FB, Zelman KM, Mozaffarian D, Kris-Etherton PM. A healthy approach to dietary fats: understanding the science and taking action to reduce consumer confusion. Nutr J. 2017;16(1):53. Epub 2017/09/01. 10.1186/s12937-017-0271-4 . 28854932 PMC5577766

[9] Zhang B, Zhai FY, Du SF, Popkin BM. The China Health and Nutrition Survey, 1989–2011. Obes Rev. 2014;15 Suppl 1:2–7. Epub 2013/12/18. 10.1111/obr.12119 . 24341753 PMC3869031

[10] ChinaCDC. China Health and Nutrition Survey Work Manual 2006. https://www.cpc.unc.edu/projects/china/data/questionnaires/Training_Manual_2006_short.pdf.

[11] Yang Y, Wang GY, Pan XC. Chinese Food Composition Table 2002. China: Beijing Medical University; 2002 12 2002.

[12] Yang Y. Chinese Food Composition Table 2004. China: Beijing Medical University Press; 2004 5 1, 2005.

[13] Shen X, Fang A, He J, Liu Z, Guo M, Gao R, et al. Trends in dietary fat and fatty acid intakes and related food sources among Chinese adults: a longitudinal study from the China Health and Nutrition Survey (1997–2011). Public Health Nutr. 2017;20(16):2927–36. Epub 2017/08/10. 10.1017/S1368980017001781 . 28789722 PMC10261479

[14] Committee. CEC. China overweight/obesity medical nutrition treatment expert consensus (2016 edition). Chin J Diabetes Mellitus. 2016;8(9):525–40.

[15] Czernichow S, Kengne AP, Stamatakis E, Hamer M, Batty GD. Body mass index, waist circumference and waist-hip ratio: which is the better discriminator of cardiovascular disease mortality risk?: evidence from an individual-participant meta-analysis of 82 864 participants from nine cohort studies. Obes Rev. 2011;12(9):680–7. Epub 2011/04/28. 10.1111/j.1467-789X.2011.00879.x . 21521449 PMC4170776

[16] Wang L, Manson JE, Gaziano JM, Buring JE, Sesso HD. Fruit and vegetable intake and the risk of hypertension in middle-aged and older women. Am J Hypertens. 2012;25(2):180–9. Epub 2011/10/14. 10.1038/ajh.2011.186 . 21993367 PMC3258456

[17] Borgi L, Muraki I, Satija A, Willett WC, Rimm EB, Forman JP. Fruit and Vegetable Consumption and the Incidence of Hypertension in Three Prospective Cohort Studies. Hypertension. 2016;67(2):288–93. Epub 2015/12/09. 10.1161/HYPERTENSIONAHA.115.06497 . 26644239 PMC5350612

[18] Wang SS, Lay S, Yu HN, Shen SR. Dietary Guidelines for Chinese Residents (2016): comments and comparisons. J Zhejiang Univ Sci B. 2016;17(9):649–56. Epub 2016/09/09. 10.1631/jzus.B1600341 . 27604857 PMC5018612

[19] Shah M, Adams-Huet B, Garg A. Effect of high-carbohydrate or high-cis-monounsaturated fat diets on blood pressure: a meta-analysis of intervention trials. Am J Clin Nutr. 2007;85(5):1251–6. Epub 2007/05/11. 10.1093/ajcn/85.5.1251 . 17490960

[20] Jalal DI, Smits G, Johnson RJ, Chonchol M. Increased fructose associates with elevated blood pressure. J Am Soc Nephrol. 2010;21(9):1543–9. Epub 2010/07/03. 10.1681/ASN.2009111111 . 20595676 PMC3013529

[21] Binia A, Jaeger J, Hu Y, Singh A, Zimmermann D. Daily potassium intake and sodium-to-potassium ratio in the reduction of blood pressure: a meta-analysis of randomized controlled trials. J Hypertens. 2015;33(8):1509–20. Epub 2015/06/04. 10.1097/HJH.0000000000000611 . 26039623

[22] Perez V, Chang ET. Sodium-to-potassium ratio and blood pressure, hypertension, and related factors. Adv Nutr. 2014;5(6):712–41. Epub 2014/11/16. 10.3945/an.114.006783 . 25398734 PMC4224208

[23] Zhai FY, Du SF, Wang ZH, Zhang JG, Du WW, Popkin BM. Dynamics of the Chinese diet and the role of urbanicity, 1991–2011. Obes Rev. 2014;15 Suppl 1:16–26. Epub 2013/12/18. 10.1111/obr.12124 . 24341755 PMC3868998

[24] Engberink MF, Hendriksen MA, Schouten EG, van Rooij FJ, Hofman A, Witteman JC, et al. Inverse association between dairy intake and hypertension: the Rotterdam Study. Am J Clin Nutr. 2009;89(6):1877–83. Epub 2009/04/17. 10.3945/ajcn.2008.27064 . 19369377

[25] Lee SA, Cai H, Yang G, Xu WH, Zheng W, Li H, et al. Dietary patterns and blood pressure among middle-aged and elderly Chinese men in Shanghai. Br J Nutr. 2010;104(2):265–75. Epub 2010/03/02. 10.1017/S0007114510000383 . 20187997 PMC2904427

[26] Appleby PN, Davey GK, Key TJ. Hypertension and blood pressure among meat eaters, fish eaters, vegetarians and vegans in EPIC-Oxford. Public Health Nutr. 2002;5(5):645–54. Epub 2002/10/10. 10.1079/PHN2002332 . 12372158

[27] Wang Y, Wang L, Xue H, Qu W. A Review of the Growth of the Fast Food Industry in China and Its Potential Impact on Obesity. Int J Environ Res Public Health. 2016;13(11). Epub 2016/11/12. 10.3390/ijerph13111112 . 27834887 PMC5129322

[28] Alter DA, Eny K. The relationship between the supply of fast-food chains and cardiovascular outcomes. Can J Public Health. 2005;96(3):173–7. Epub 2005/05/26. 10.1007/BF03403684 . 15913078 PMC6976214

[29] Kar S, Khandelwal B. Fast foods and physical inactivity are risk factors for obesity and hypertension among adolescent school children in east district of Sikkim, India. J Nat Sci Biol Med. 2015;6(2):356–9. Epub 2015/08/19. 10.4103/0976-9668.160004 . 26283829 PMC4518409

[30] Xu H, Short SE, Liu T. Dynamic relations between fast-food restaurant and body weight status: a longitudinal and multilevel analysis of Chinese adults. J Epidemiol Community Health. 2013;67(3):271–9. Epub 2012/08/28. 10.1136/jech-2012-201157 . 22923769 PMC3574174

[31] Sacks FM, Svetkey LP, Vollmer WM, Appel LJ, Bray GA, Harsha D, et al. Effects on blood pressure of reduced dietary sodium and the Dietary Approaches to Stop Hypertension (DASH) diet. DASH-Sodium Collaborative Research Group. N Engl J Med. 2001;344(1):3–10. Epub 2001/01/04. 10.1056/NEJM200101043440101 . 11136953

[32] Bray GA, Vollmer WM, Sacks FM, Obarzanek E, Svetkey LP, Appel LJ, et al. A further subgroup analysis of the effects of the DASH diet and three dietary sodium levels on blood pressure: results of the DASH-Sodium Trial. Am J Cardiol. 2004;94(2):222–7. Epub 2004/07/13. 10.1016/j.amjcard.2004.03.070 . 15246908

[33] Liu Z. Dietary sodium and the incidence of hypertension in the Chinese population: a review of nationwide surveys. Am J Hypertens. 2009;22(9):929–33. Epub 2009/08/08. 10.1038/ajh.2009.134 . 19661928

[34] Poslusna K, Ruprich J, de Vries JH, Jakubikova M, van’t Veer P. Misreporting of energy and micronutrient intake estimated by food records and 24 hour recalls, control and adjustment methods in practice. Br J Nutr. 2009;101 Suppl 2:S73–85. Epub 2009/07/15. 10.1017/S0007114509990602 . 19594967

[35] FAO. Dietary assessment: A resource guide to method selection and application in low resource settings: FAO; 2018.

