# Supplementary material for: Impact of different dietary fat sources on blood pressure in Chinese adults
Source: PLoS One. 2021 Mar 8;16(3):e0247116. doi: 10.1371/journal.pone.0247116 (PMC7939266; doi:10.1371/journal.pone.0247116)
Supplement: S1 File — (DOCX) [file pone.0247116.s001.docx]

**S1 File**

**Calculation on an individual’s percentage of dietary fat intake from a food type**

In China Health and Nutrition Survey (CHNS), each food in an individual’s 24-hour recall dietary assessment was identified, categorized, and coded using China Food Composition Table (FCT) 2002 and 2004. The food code and food type are unique for each food. A food type can be assigned to different foods.

In FCT, each coded food has its fat per 100 edible grams listed. Therefore, by matching a food’s food code and food type in CHNS with the code and type in FCT, we obtained the amount of fat per 100 edible grams of each recorded food in CHNS.

We designed the following formulas:

An individual’s fat intake from a given food Z is calculated as:

$${DFI}_{Z}=\frac{I_{Z}}{100}\times{EP}_{Z}\times F_{Z} (1)$$

where:

*DFI_Z_* = Dietary fat intake from food Z in grams

*Z=* Food Z

*I_Z_* = Intake amount of food Z in gram as recorded in CHNS

*EP_Z_* = Edible portion of food Z per 100 grams as listed in FCT, in percentage

*F*_Z_= Fat content of food Z per 100 edible grams as listed in FCT

It is common for an individual to consume several foods of the same food type in 24 hours. E.g., in terms of meat, an individual can consume pork in the morning, beef at noon, and lamb in the evening. With the individual’s fat intake from a food calculated by *Formula (1)*, we aggregated the fat intake from all the foods of the same food type to get an individual’s total intake of fat obtained from a food type.

An individual’s total intake of fat from food type *T* is calculated as:

$${TFI}_{T}={DFI}_{\left( k1,T \right)}+{DFI}_{(k2,T)}+\ldots+{DFI}_{\left( kn,T \right)} (2)$$

where:

*TFI_T_ =*Total fat intake from food type T

*T=* Food type T

*k1, k2…kn, =* Food consumed by the individual, all the food are of food type T

*DFI_(k1,T)_ =* An individual’s fat intake from food *k1* with food type T

With the individual’s fat intake from food type T calculated by *Formula (2)*, we divided the fat intake from food type T by the individual’s total fat intake to get the percentage of the fat intake from food type T in relation to an individual’s total fat intake.

The percentage of an individual’s fat intake from food type T in relation to the individual’s total fat intake is calculated as:

$${PTFI}_{T}=\frac{{TFI}_{T}}{Sum of the individual^{'}s fat intake from all the foods}\times100\% (3)$$

where:

*PTFI_T_* = Percentage of an individual’s total fat intake from food type T

*TFI_T_ =* An individual’s fat intake from food type T
